# Zoomed MRI Guided by Combined EEG/MEG Source Analysis: A Multimodal Approach for Optimizing Presurgical Epilepsy Work-up and its Application in a Multi-focal Epilepsy Patient Case Study

**DOI:** 10.1007/s10548-017-0568-9

**Published:** 2017-05-16

**Authors:** Ü. Aydin, S. Rampp, A. Wollbrink, H. Kugel, J. -H. Cho, T. R. Knösche, C. Grova, J. Wellmer, C. H. Wolters

**Affiliations:** 1 Institute for Biomagnetism und Biosignalanalysis, University of Münster, Malmedyweg 15, 48149 Münster, Germany; 2 Multimodal Functional Imaging Lab, Department of Physics and PERFORM Centre, Concordia University, Montreal, Quebec, Canada; 3 Department of Neurosurgery, University Hospital Erlangen, Erlangen, Germany; 4 Department of Clinical Radiology, University Hospital Münster, Münster, Germany; 5 Max Planck Institute for Human Cognitive and Brain Sciences, Leipzig, Germany; 6 Multimodal Functional Imaging Lab, Department of Biomedical Engineering, McGill University, Montreal, Quebec, Canada; 7 Department of Neurology and Neurosurgery, Montreal Neurological Institute, McGill University, Montreal, Quebec, Canada; 8 Ruhr-Epileptology, Department of Neurology, University Hospital Knappschaftskrankenhaus Bochum, Bochum, Germany

**Keywords:** Combined EEG/MEG, Zoomed MRI, Epileptic activity, Source reconstruction, Realistic finite element head model, Skull conductivity calibration, Focal cortical dysplasia type IIb

## Abstract

In recent years, the use of source analysis based on electroencephalography (EEG) and magnetoencephalography (MEG) has gained considerable attention in presurgical epilepsy diagnosis. However, in many cases the source analysis alone is not used to tailor surgery unless the findings are confirmed by lesions, such as, e.g., cortical malformations in MRI. For many patients, the histology of tissue resected from MRI negative epilepsy shows small lesions, which indicates the need for more sensitive MR sequences. In this paper, we describe a technique to maximize the synergy between combined EEG/MEG (EMEG) source analysis and high resolution MRI. The procedure has three main steps: (1) construction of a detailed and calibrated finite element head model that considers the variation of individual skull conductivities and white matter anisotropy, (2) EMEG source analysis performed on averaged interictal epileptic discharges (IED), (3) high resolution (0.5 mm) zoomed MR imaging, limited to small areas centered at the EMEG source locations. The proposed new diagnosis procedure was then applied in a particularly challenging case of an epilepsy patient: EMEG analysis at the peak of the IED coincided with a right frontal focal cortical dysplasia (FCD), which had been detected at standard 1 mm resolution MRI. Of higher interest, zoomed MR imaging (applying parallel transmission, ‘ZOOMit’) guided by EMEG at the spike onset revealed a second, fairly subtle, FCD in the left fronto-central region. The evaluation revealed that this second FCD, which had not been detectable with standard 1 mm resolution, was the trigger of the seizures.

## Introduction

Despite considerable advancements in electroencephalography (EEG) and magnetoencephalography (MEG) source analysis, these techniques’ sensitivity, specificity, and spatial resolution to completely replace invasive recordings are still under discussion. In many cases, source analysis is used only to guide placement of depth EEG-electrodes. Although knowing the exact region to implement invasive electrodes is very critical, the ultimate aim of EEG/MEG source analysis is to minimize the necessity for invasive recordings. This could lead to important benefits by avoiding the complications of invasive recording procedures (Hamer et al. 2002; Wellmer et al. 2012). Furthermore, invasive recordings can only measure activity within a close distance from the sensors, suffer from low spatial sampling due to limited numbers of invasive electrodes, and exhibit a tunnel view effect due to limited coverage (Lüders et al. 2006).

Similar to invasive recordings, EEG and MEG have high temporal resolution in the range of milliseconds (ms) and measure the electrical activity of neurons directly without using indirect phenomena like hemodynamics or metabolism, which is the case for functional magnetic resonance imaging (fMRI) or positron emission tomography (PET). However, the spatial resolutions of EEG and MEG are lower in comparison to fMRI and their sensitivities decrease with the distance between sources and sensors. EEG and MEG source analysis bears some specific challenges. Some of these are related to the forward problem: developing methodology and pipelines to model the head and the brain as accurate as possible, while still keeping the setup time and computational costs at a reasonable level for clinical use. Although there has been considerable advancement in this area, especially within the finite element framework (Wolters et al. 2002; Rullmann et al. 2009; Vorwerk et al. 2014), three compartment models calculated with the boundary element method are still the most widely deployed ones in the field. The main difficulty related to the inverse problem of EEG and MEG is its non-uniqueness. This means that there is, without further prior information, an infinite number of source configurations that results in the same EEG/MEG signals (Hämäläinen et al. 1993). Many promising inverse approaches have been developed to alleviate this problem by using different constraints (Pascual-Marqui 2002; Lucka et al. 2012; Chowdhury et al. 2013; Lina et al. 2014). Instead of solving the EEG and MEG inverse problems independently, performing combined EEG/MEG (EMEG) source analysis leads to significant improvements (Aydin et al. 2015; Chowdhury et al. 2015). These improvements are particularly significant for scenarios with low signal-to-noise ratio (SNR), such as for deep sources or at the spike onset (Aydin et al. 2015).

The specificity of EEG, MEG and EMEG source analysis could be significantly increased by incorporating other available information. The additional information might come from other functional imaging techniques such as fMRI or PET, from seizure semiology, or from MRI sequences sensitive to structural changes and lesions. In general, not every lesion evident in structural MRI is related to the epilepsy, but some types of lesions, such as focal cortical dysplasia (FCD) type IIB are shown to be highly epileptogenic (Wagner et al. 2011). Furthermore, it has been reported that in up to 73% of MRI negative cases, histology shows an underlying FCD (Lee et al. 2005). Therefore, reinvestigating structural MRI by incorporating the findings from source analysis may be a beneficial practice in presurgical epilepsy diagnosis (Moore et al. 2002; Wang et al. 2012).

In this study, we introduce a pipeline that combines information from EMEG source analysis, seizure semiology, and high resolution structural MRI in presurgical epilepsy diagnosis. We constructed a high resolution (1 mm edge length) head model that distinguishes seven different tissue types, uses diffusion tensor imaging (DTI) to amend the anisotropic white matter compartment, and benefits from a calibration procedure to estimate individual skull conductivity. This head model was then used to solve the forward problem with the finite element method (Aydin et al. 2014) and perform EMEG source analysis (Aydin et al. 2015). The most important novelty of this paper is coupling EMEG source analysis with a ‘zoomed’ MRI sequence that allows localized excitation utilizing parallel transmission (ZOOMit) (Blasche et al. 2012). This technique is capable of acquiring data with 0.5 mm voxel edge length of a restricted area within a reasonable time. By combining EMEG source analysis, seizure semiology information, and the ZOOMit MRI, a subtle FCD, which was undetectable at the lower resolution (1 mm), was detected near the epileptic focus localized by EMEG. To the best of our knowledge this is the first study that combines source analysis and zoomed MRI in the field of epilepsy.

## Patient and Methods

### Ethics Statement

The patient gave her written informed consent and all procedures have been approved by the ethics committee of the University of Erlangen, Faculty of Medicine on 10.05.2011 (Ref. No. 4453).

### Patient

**Fig. 1:**
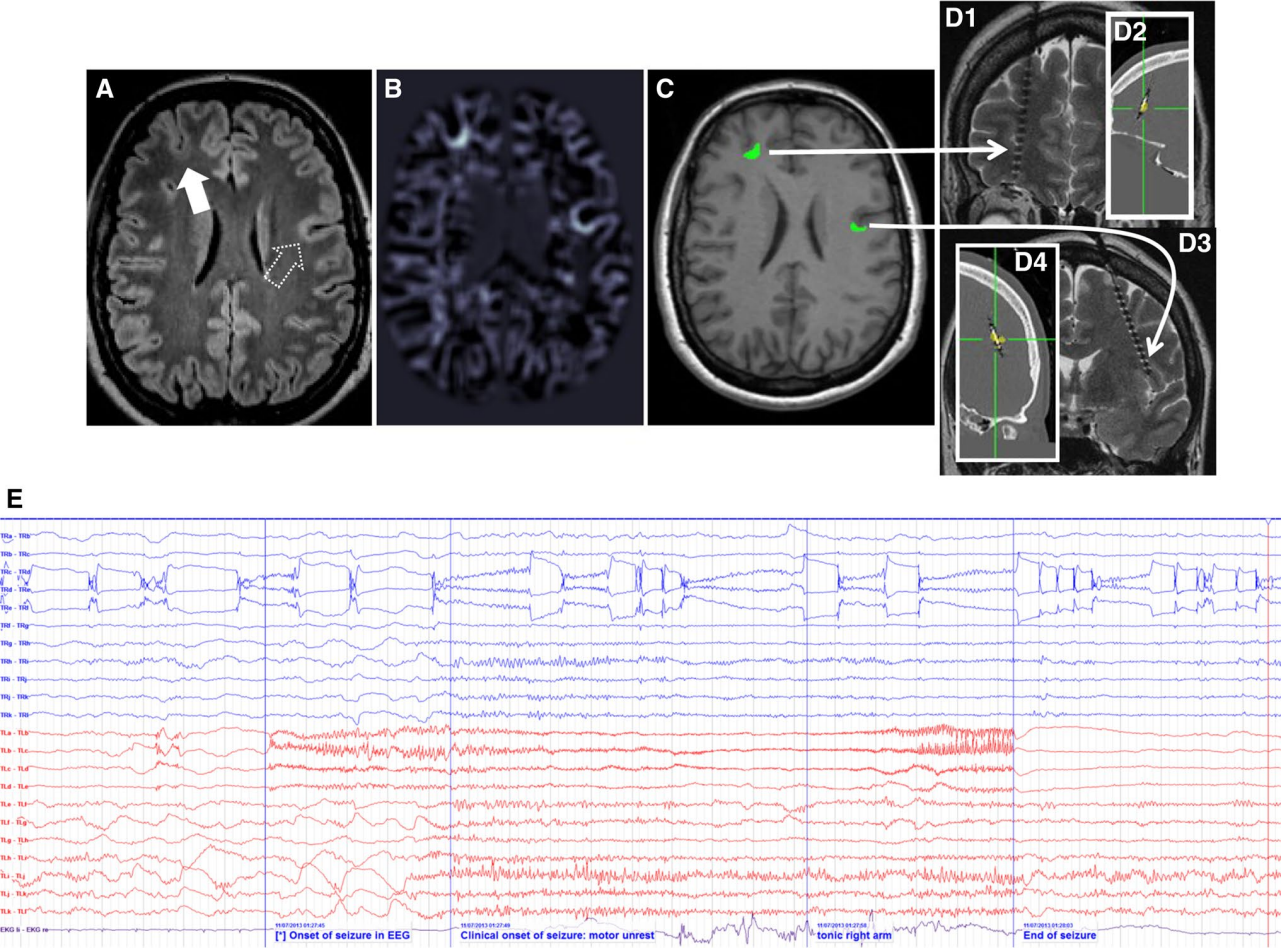
Clinical work-up of the patient. *A* Diagnostic MRI at 3 T (including 3D-FLAIR with 1 mm voxel edge length) showed the right frontal FCD IIB (*full arrow*) but was negative for the second FCD (*dotted arrow*). Morphometric MRI-analysis (*B*) led to a suspicion of the existence of two FCDs in the junction analysis. After transforming the junction analysis abnormalities into ROIs (*C*) a minimal invasive, confirmative implantation strategy was chosen to document interictal activity and seizure onset in either suspected FCD. *Panel D* shows the ROI-based implantation of each one depth electrode into the right and left frontal FCD (*D1* MRI documentation of the right frontal depth electrode; *D2* according to overlay with CT the depth electrodes penetrate the ROI perfectly; *D3* and *4*: same for the left frontal FCD). Note radiological convention on MR images: patient’s right is viewer’s left. Interictal EEG showed the typical discharge pattern often seen in FCD IIB in both lesions. Seizure onset, however, was documented only in the left FCD IIB. *Panel E*: *blue traces* represent the right frontal FCD, *red traces* the left frontal FCD, *traces 1–3* represent the intralesional contacts; note that the short seizure is running in the left frontal FCD while the interictal discharge pattern in the right frontal FCD continues unaffected. (Color figure online)

Data was acquired from a 49-year-old female suffering from pharmaco-resistant focal onset epilepsy since the second year of life. The patient used 8 life-time antiepileptic drugs, but was still suffering from 100 to 200 seizures per month. The seizure semiology, involving tingling feeling at the right anterior torso, ascending feeling of nausea, then loss of consciousness and tonic or hypermotor movement of right arm and leg, was pointing to left frontocentral regions. Discordantly, diagnostic MRI revealed a right frontal FCD on a 3D-FLAIR sequence (with a resolution of 1 mm^3^) prior to source analysis. Morphometric MRI analysis (Huppertz et al. 2005) results were not very clear, but led to a suspicion of a possible second left frontal focal cortical dysplasia (Fig. 1b). However, visual reinspection of the MRI (3D-FLAIR at 3 T with voxels of 1 × 1 × 1 mm^3^) could not confirm this suspicion (Fig. 1a).

Interictal and ictal surface EEG (standard 10/20 montage) did not show reproducible epileptic discharges. FDG-PET failed to reveal a focal hypometabolism at the sites of the two suspected FCD, only left temporo-mesial structures showed a little less glucose uptake than contralateral. Because of non temporo-mesial seizure semiology this was regarded as unspecific. For intracranial EEG recordings each one depth electrode (AD-tech, Racine, WI U.S.A) was stereotactically inserted into the regions-of-interest derived from morphometric MRI-analysis (MRIcro (Chris Rorden, Version 1.37) imported into Iplan software, Brainlab, Feldkirchen, Germany; Wellmer et al. 2010) (Fig. 1c, d). The recording of strong interictal epileptic discharge activity confirmed the suspicion of FCD IIB in both localizations. Seizure onset, however, was documented only in the left hemispheric lesion—few seconds before clinical seizure onset (Fig. 1e). Responsibility of the left FCD IIB was further confirmed by the result of surgical treatment of the left, but not the right frontal FCD. Following stereotactic, lesion focused radiofrequency thermocoagulation the patient had a truncation of symptoms of her seizures (postoperatively only short arousal tonic and right arm but no more hypermotor seizure component). The most likely reason for failure to achieve complete seizure freedom is that the coagulation missed a small part of the lesion (for more details see discussion in Wellmer et al. 2016).

### MR Acquisitions

**Fig. 2:**
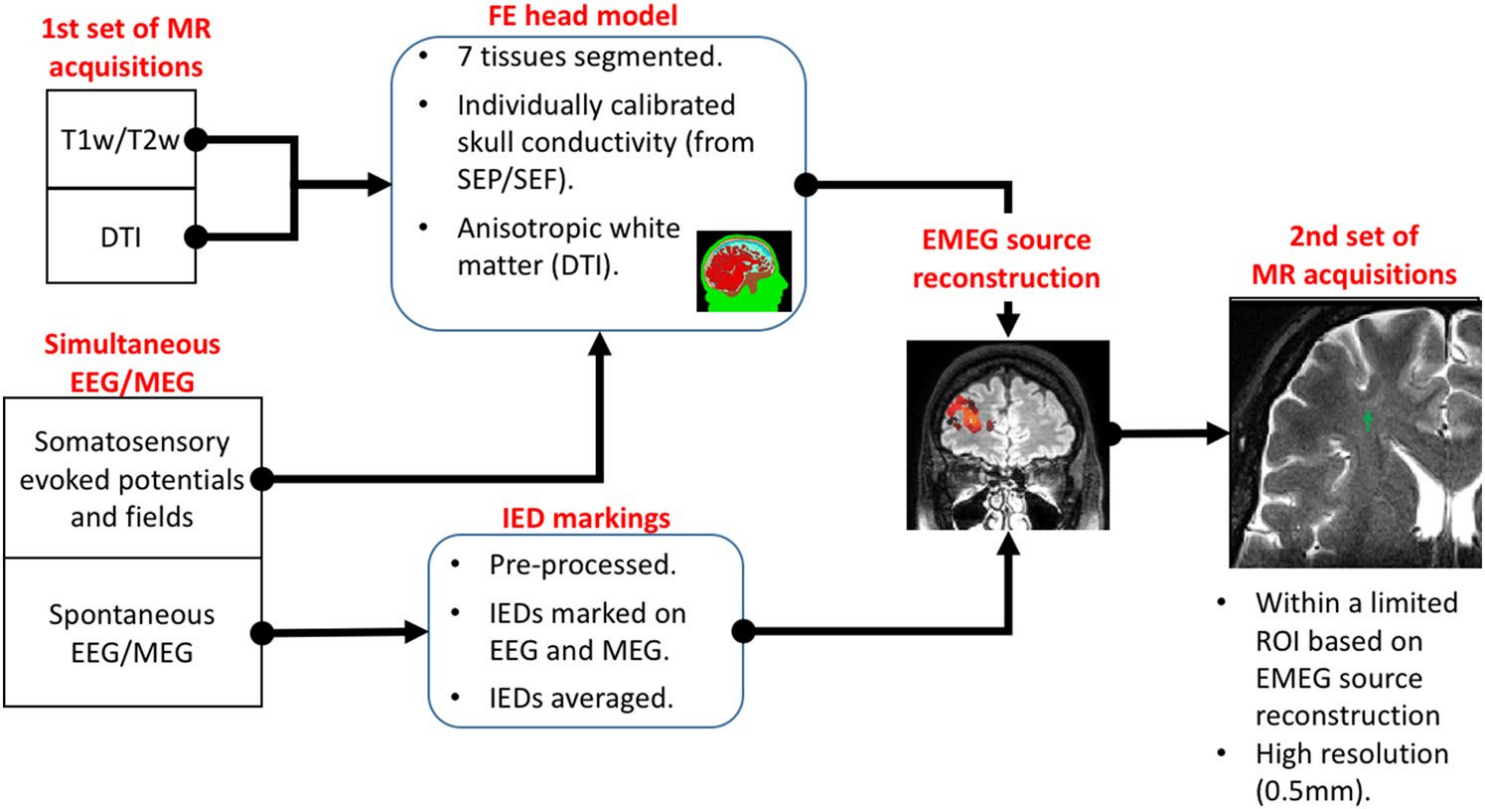
Scheme of the analysis strategy

In addition to the diagnostic 3 T MRI, two sets of MRI data were acquired on two different 3 T scanners. The first scans were acquired prior to EEG/MEG source analysis for the main purpose of building the head model and finding cortical malformations. The second scan was done at a later date and was guided by the source analysis results (see Fig. 2 for a scheme of the analysis strategy). Note the radiological convention of left and right in all presented MR images (patient’s left is viewer’s right).

#### First Study MR Acquisition (Prior to Source Analysis)

A 3 T scanner (Gyroscan Intera/Achieva 3.0 T, System Release 2.5 (Philips Healthcare, Best, NL)) was used for the acquisition. The specific sequences were:

1. 3D-T1-weighted (T1w) fast gradient-echo pulse sequence (TFE) using water selective excitation to avoid shifted fat signal (TR/TE/FA = 9.2/4.4 ms/9°, inversion prepulse every 1015.5 ms, cubic voxels of 1.17 mm edge length).
2. 3D-T2w turbo spin echo pulse sequence (TR/TE = 2000/378 ms, cubic voxels, 1.17 mm edge length).
3. Diffusion tensor (DT) MRI using an echo planar imaging sequence (Stejskal-Tanner spin-echo, TR/TE = 7546/67 ms, cubic voxels, 1.875 mm edge length), with one volume with diffusion sensitivity b = 0 s/mm^2^ (i.e., flat diffusion gradient) and 20 volumes with b = 1000 s/mm^2^ in different directions, equally distributed on a sphere. Another volume with flat diffusion gradient, but with reversed spatial encoding gradients was acquired and used for susceptibility artifact correction (Ruthotto et al. 2012).
4. 3D-FLAIR (Fluid-attenuated inversion recovery, TR/TE = 7000/322 ms, inversion time 2400 ms, cubic voxels, 1.17 mm edge length). This sequence was used to detect possible cortical malformations and lesions.

The acquisition times required for each of these four scans were approximately 7 min. In order to improve the co-registration between MRI and EEG/MEG electrodes/sensors, three gadolinium filled markers were placed on the nasion as well as inside the left and right ear canals prior to the MRI scan.

#### Second Study MR Acquisition (Guided by Source Analysis)

A second set of MRI data was acquired with another 3 T scanner (MAGNETOM Prisma 3.0 T, Release D13 [Siemens Medical Solutions, Erlangen, Germany]). The main reason for selecting another scanner for this part of the examination was to benefit from a novel MRI technique that employs localized excitation utilizing 2D selective RF pulses (Finsterbusch 2010) with parallel transmission (ZOOMit) (Blasche et al. 2012). Localized excitation allows to ‘zoom’ a field of view, restricting excitation to a desired area even within brain tissue without aliasing artifacts that occur when the FOV is smaller than the imaged object. This avoids the need to increase the number of phase encoding steps and the penalty of an increased minimum measurement time. The number of phase encoding steps necessary to obtain sufficient signal to noise can be used to increase the acquired volume in slice encoding direction, allowing a flexible definition of the volume of interest for searching the lesion in three dimensions. In the case presented here the acquired volume was a cuboid of 160 mm × 82 mm × 28 mm (lr × ap × fh, frequency × phase × slice encoding (2nd phase encoding)) with cubic voxels of 0.5 mm × 0.5 mm × 0.5 mm edge length.

Lesion visibility was additionally improved by the choice of contrast parameters. Our setting with (nominal) TR/TE/TI 2320/198/1800 ms in a 3D Turbo-Inversion Recovery technique with Flip angle control (SPACE) resulted in a combination of T2- and T1-weighting with sufficient signal strength to show the FCD. The acquisition time was about 13 min. Two different regions of interest (ROIs) at right frontal and left frontocentral locations were selected based on the findings of EMEG source analysis (explained in detail in the following sections).

### Electrophysiological Measurements

EEG, MEG and ECG were recorded simultaneously in a magnetically shielded room. The EEG cap had 80 AgCl sintered ring electrodes (EASYCAP GmbH, Herrsching, Germany). The MEG was acquired with a whole head system with 275 axial gradiometers and 29 reference coils (OMEGA2005, CTF, VSM MedTech Ltd., Canada). The reference coils were used to calculate 3rd order synthetic gradiometers, thereby reducing the interference of magnetic fields originating from distant locations (e.g., Magnetocardiogram).

The patient was measured in supine position to reduce head movements and to avoid brain shift. Rice and colleagues (Rice et al. 2013) have shown that brain shift results in changes in CSF thickness and even these small changes affect EEG signals with 80% power difference on average due to the high conductivity of CSF.

The electrode positions were digitized with a Polhemus device (FASTRAK^®^, Polhemus Incorporated, Colchester, Vermont, U.S.A.) prior to the measurement. During the recordings the position of the head inside the MEG scanner was constantly measured via three head localization coils placed on the nasion and in the ear canals (same positions as the gadolinium markers in MRI).

In total seven runs were acquired. During the first run (7 min long) the median nerve of the right arm of the patient was stimulated with electrical pulses just above the motor threshold (used to calibrate skull conductivity for the head model, see (Aydin et al. 2014) for details). This run was followed by six 8 min long runs (2400 Hz sampling rate, low pass filtered at 600 Hz), in which the patient was advised to relax and close her eyes, aimed at measuring interictal epileptic discharges.

### Calibrated Finite Element Head Model and Forward Solution

**Fig. 3:**
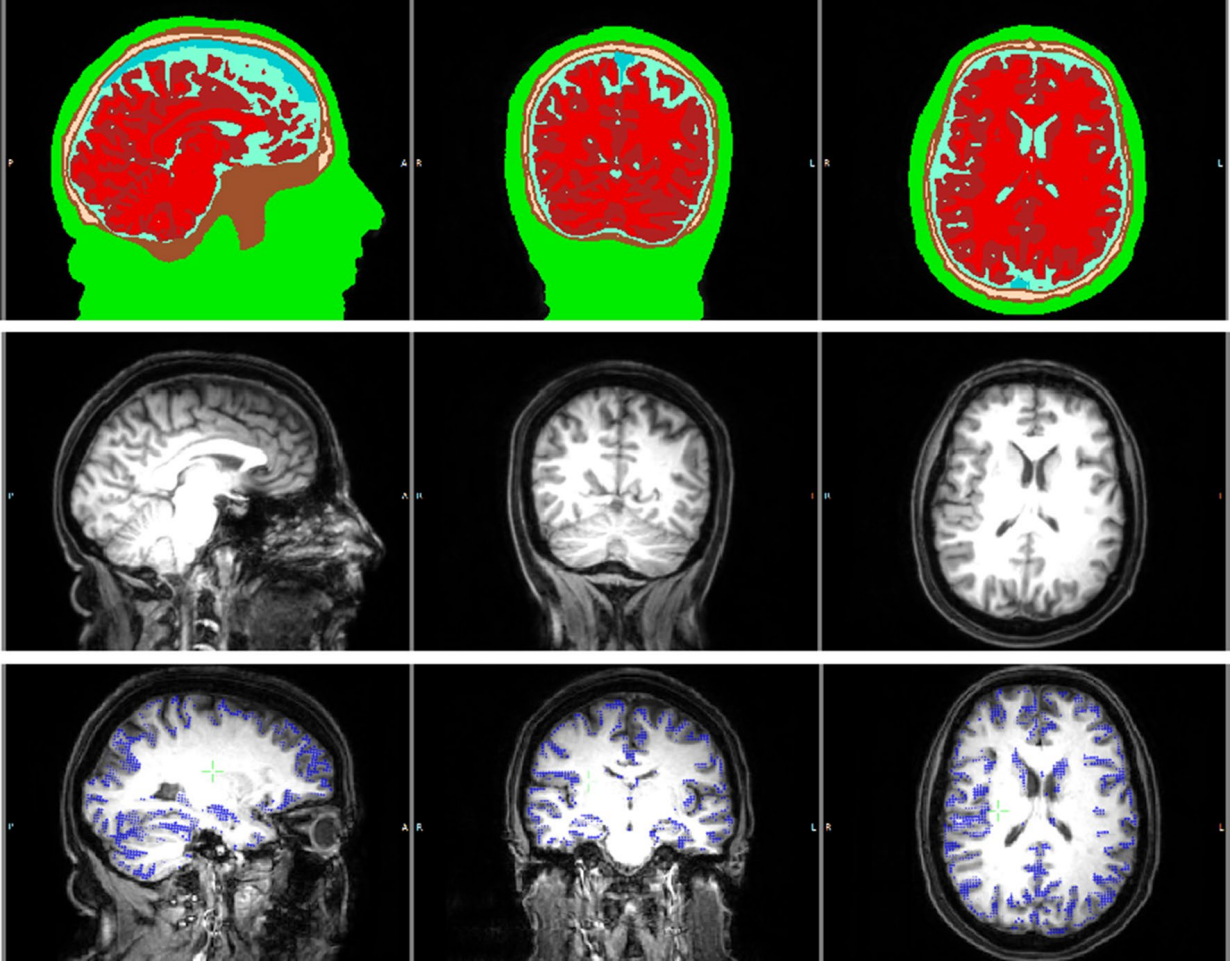
Segmented MRI (*upper row*), T1w MRI (*middle row*) and the source space points (*blue points*) shown on T1w (*lower row*) MRI. Sagittal (*left column*), coronal (*middle column*) and axial (*right column*) slices are shown. Please note that the slices selected in the lower row are different from the top two rows in order to better visualize the source space points. The color codes for the tissues in the segmented MRI are scalp (*green*), skull compacta (*brown*), skull spongiosa (*beige*), dura mater (*dark turquoise*), CSF (*light turquoise*), gray matter (burgundy) and white matter (*red*). *White letters* on the MRIs show the directions (*L* left, *R* right, *A* anterior, *P* posterior). (Color figure online)

T1w and T2w MRIs were used to construct an individual seven-compartment head model that distinguishes scalp, skull spongiosa, skull compacta, dura mater, cerebrospinal fluid (CSF), gray matter (GM), and white matter (WM). The resulting segmentation and T1w MRI are shown in Fig. 3 upper and middle rows, respectively. The pipeline used for registration and segmentation was similar to the one explained before (Aydin et al. 2014), however, following recent findings (Ramon 2012; Ramon et al. 2014; Fiederer et al. 2015), an additional tissue type, the dura mater, was segmented and included as the seventh compartment. The segmentation of the dura mater, including the sagittal sinus space filled with venous blood that is surrounded by dura mater, was performed using Seg3D^1^ and involved manual segmentation as well as some basic image processing steps such as smoothing and thresholding.

Diffusion tensors were calculated from the individual DTI data and used to model the WM conductivity tensors by an effective medium approach (Tuch et al. 2001; Rullmann et al. 2009; Ruthotto et al. 2012; Aydin et al. 2014). These conductivity tensors were later included into the head model to account for the anisotropic WM tissue.

The importance of skull conductivity has been shown for EEG and MEG source reconstruction in adults (Aydin et al. 2014) as well as for EEG in neonates (Roche-Labarbe et al. 2008). The conductivity of skull shows a high inter- and intra-individual variance. EEG source reconstructions are strongly influenced by changes in skull conductivity, while these effects are considerably smaller for MEG. Therefore, in (Aydin et al. 2014, see algorithm 2) we used a dipole scanning strategy that benefits from the different sensitivity profiles of EEG and MEG to calibrate skull conductivity. The calibration procedure used in this work could be summarized as first benefitting from the low sensitivity of MEG to skull conductivity and localizing the primary somatosensory cortex even for less suitable skull conductivity parameters. Then, fixing the location and determining the orientation of the dipole with EEG (to compensate for the insensitivity of MEG to quasi-radial source components). Finally, determining the appropriate skull conductivity by comparing the magnitudes of EEG and MEG dipoles for this fixed position and orientation. We have used the somatosensory P20/N20 response for the calibration procedure because it is well known that the generators of this component are localized in Brodmann area 3b and are focal, not too deep and mainly tangentially oriented (Allison et al. 1991). Details of the skull calibration procedure used in this study can be found in (Aydin et al. 2014). The calibrated conductivities were calculated as 0.0033 S/m for skull compacta and 0.0116 S/m for skull spongiosa. Other tissue conductivities (S/m) used in this study were: scalp (0.43) (Ramon et al. 2004), CSF (1.79) (Baumann et al. 1997), GM (0.33) (Fuchs et al. 1998), dura mater (0.1) (Ramon 2012).

A geometry adapted hexahedral finite element mesh was created out of the segmented MRI using SimBio-VGRID^2^. Geometry adapted hexahedral meshes provide a good balance by achieving better conformance to the geometry than regular hexahedral meshes whilst being less time-consuming and complicated than constructing tissue-surface based conforming tetrahedral meshes (Camacho et al. 1997; Wolters et al. 2007; Wagner et al. 2016).

The source space nodes were restricted to being located inside the GM without any orientation constraint. The source singularity was modeled with the Venant direct approach (Buchner et al. 1997; Wolters et al. 2007). To satisfy the Venant condition, for each source space node, it was checked whether the adjacent FE mesh nodes belong to elements which were labeled as GM (Vorwerk et al. 2012). The final source space had an average resolution of 2 mm (see blue points in Fig. 3 bottom row).

The finite element transfer matrix approach and the algebraic multigrid preconditioned conjugate gradient (AMG-CG) solver were used for increased computational efficiency (Wolters et al. 2002, 2004). The forward solution was calculated with piecewise trilinear basis functions using the SimBio^3^ software.

### Interictal Epileptic Discharges and Source Reconstruction

**Fig. 4:**
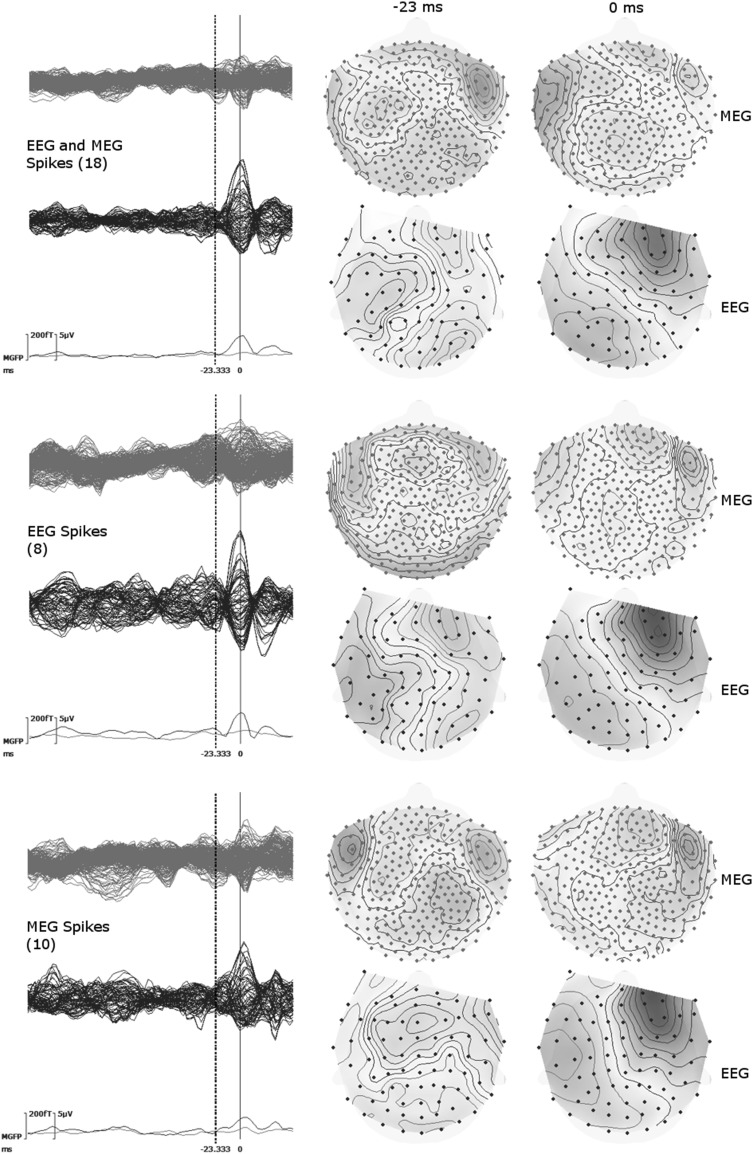
Butterfly plots for the averaged signals of spikes marked on EEG or MEG (*upper*), only from EEG (*middle row*), and only from MEG (*bottom row*). The numbers of spikes averaged for each group are given in parentheses. In butterfly plots (*left column*) MEG is shown by *green* and EEG by *blue lines*. MGFP stands for mean global field power and is given for EEG and MEG. The topographies are shown at 0 ms (close to the peak of the spike) and −23 ms (the preceding peak on MEG) as indicated by *dashed lines* on the butterfly plots. (Color figure online)

An experienced epileptologist (author SR) reviewed EEG and MEG traces separately, and marked 18 interictal epileptic discharges (IEDs). Eight of these IEDs were marked as EEG IEDs (activity more pronounced in EEG compared to MEG) with maximum negativity at F6 (will be called Espikes). Ten IEDs were marked as MEG only IEDs (Mspikes). Figure 4 shows averaged EEG and MEG signals as butterfly plots based on all IEDs (EMspikes) (top row), on Espikes only (middle row), and on Mspikes only (bottom row).

A current density approach, standardized low resolution brain electromagnetic tomography (sLORETA), was selected for source analysis (Pascual-Marqui 2002). sLORETA is a widely used source analysis method and it has been shown to perform well in situations in which multiple sources need to be accurately localized, which are temporally disentangled or whose leadfields are sufficiently uncorrelated (Pascual-Marqui 2002; Dümpelmann et al. 2012; Lucka et al. 2012). The leadfield matrices calculated with SimBio and source space points calculated with custom written Matlab code were imported into the CURRY 7^4^ software in order to solve the inverse problem.

### DTI Tractography

After performing eddy current and susceptibility corrections by following the procedure explained elsewhere (Ruthotto et al. 2012; Aydin et al. 2014), the *FSL-BEDPOSTX* routine was used to calculate the distribution of diffusion parameters at each voxel using Markov Chain Monte Carlo sampling. Afterwards, the *FSL-PROBTRACKX* function was used to perform probabilistic tractography between two ROIs (Behrens et al. 2007). These ROIs were selected based on EMEG source analysis and the subsequent ZOOMit data.

## Results

### EEG/MEG Signals and Topographies:

In this work, the peak of the EEG signal was considered as 0 ms. However, following Lantz et al. (2003) and Aydin et al. (2015) we rather applied source analysis at the middle of the rising flank to avoid wrong localizations due to possible propagation patterns. Throughout this paper, we will call localizations at −7 ms as “near spike peak” localizations.

**Fig. 5:**
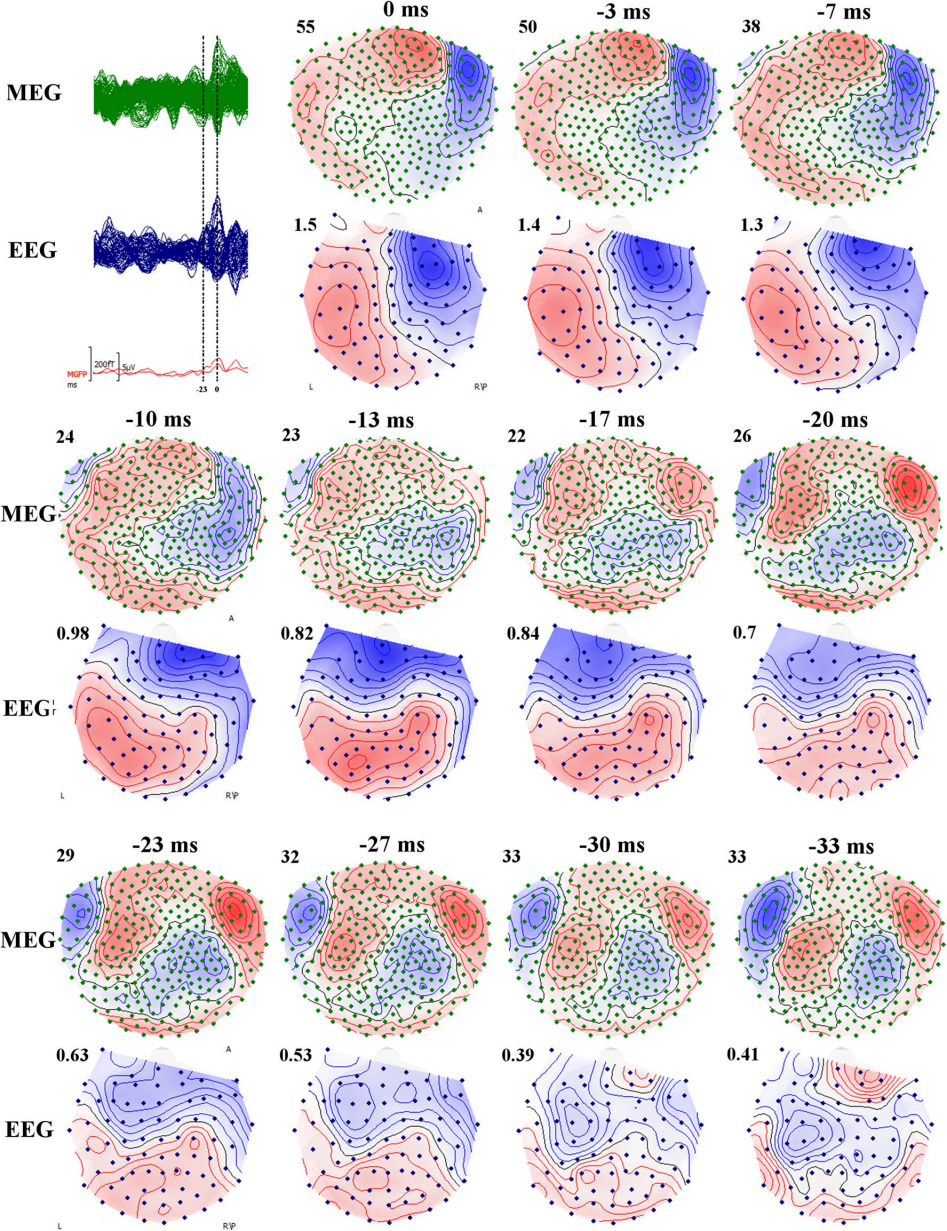
Butterfly plots (*top left*) for MEG (*green*) and EEG (*blue*), and topographies of MEG (upper topography) and EEG (bottom topography) for the averaged spike at 11 different time instances. The time points at 0 ms (peak of the spike) and −23 ms (the preceding peak on MEG) are indicated by *dashed vertical lines* in butterfly plots (*top left*). The MEG and EEG topographies are shown for every ∼3.3 ms starting from 0 ms and going backwards in time until −33 ms. In MEG and EEG topographies, the *blue* (*red*) isopotential lines indicate negativity (positivity). The increments between contour lines for each map are shown on upper left corners and the units for EEG and MEG are μV and fT respectively. *Letters* in topographies indicate the orientation (*L* left, *R* right, *A* anterior, *P* posterior). (Color figure online)

In Fig. 4, butterfly plots of EEG marked spikes (Espikes), MEG marked spikes (Mspikes), and the union of both, EMspikes, are shown. The butterfly plots of Espikes and Mspikes are different from each other (note the clearer and more distinct peak at −23 ms for Mspikes than Espikes). Both EEG and MEG topographies from Espikes and Mspikes were similar at 0 ms and indicate a dipolar pattern over the right frontal region. However, at −23 ms the topographies of Espikes and Mspikes were quite different from each other. The Mspike topographies in both MEG and EEG were pointing to another dipolar pattern over the left hemisphere, while the Espike topographies were more complicated with no clear pattern. Therefore, we will work with the Mspikes (detected from MEG) in the remaining part of the paper. Source analysis of Espikes at 0 ms gave very similar results to Mspikes but, as expected from the topographies, Espike source analysis at −23 ms was not stable. Topographies of Mspikes for other time instants could be found in Fig. 5.

### EMEG Source Analysis near Spike Peak and ZOOMit MRI of this ROI

**Fig. 6:**
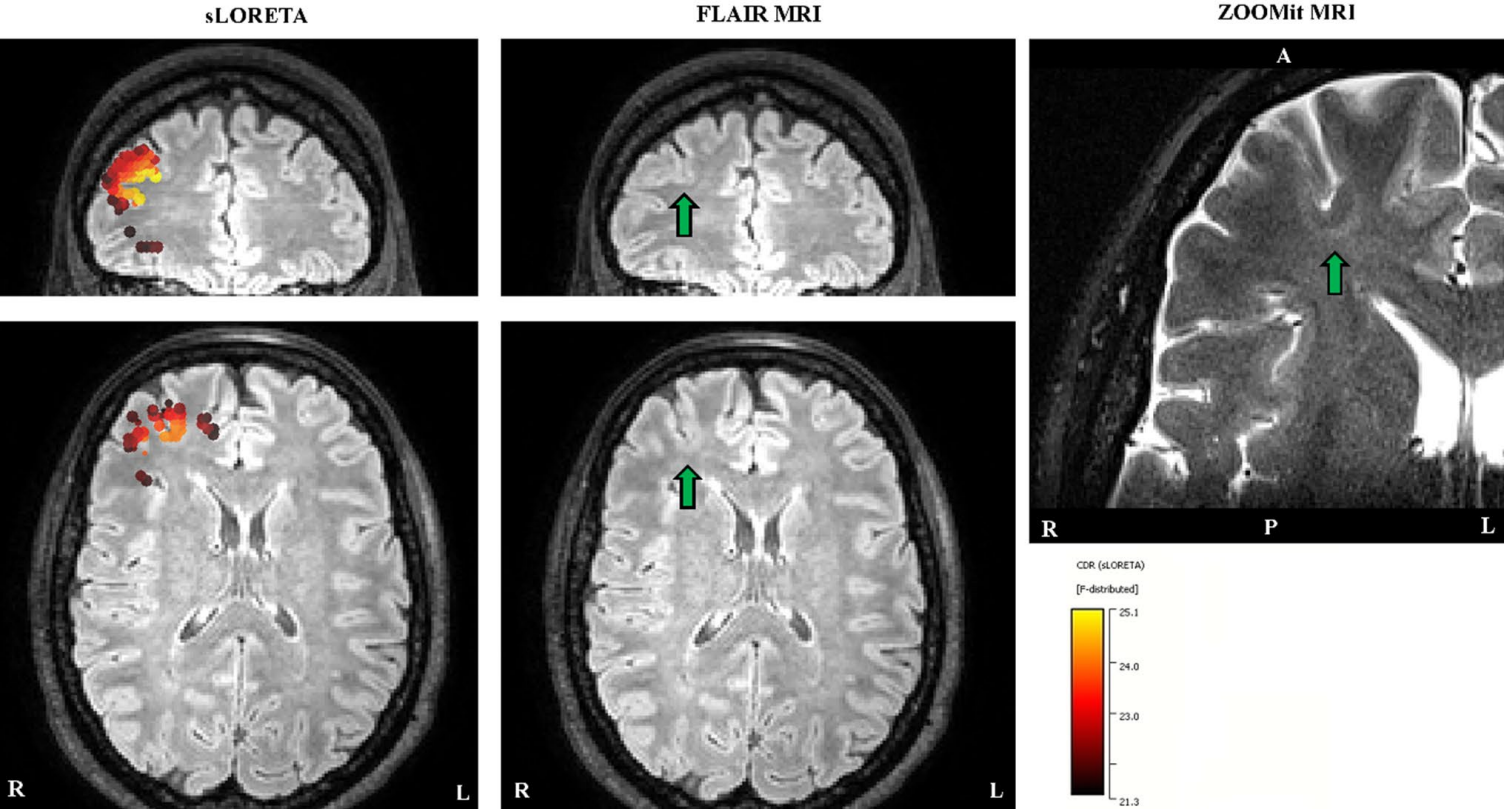
EMEG source localizations at −7 ms. In the left column the sLORETA results projected onto the FLAIR MRI are shown (only results obtained with a threshold of 85% for the maximum F-value are shown). In the middle column, the FLAIR image without the localizations is shown, in order to enable the identification of the FCDs (pointed to by *green arrows*). The right column shows the result of ZOOMit MRI with the *green arrow* indicating the FCD. *White letters* on the MRIs show the directions (*L* left, *R* right, *A* anterior, *P* posterior). (Color figure online)

**Fig. 7:**
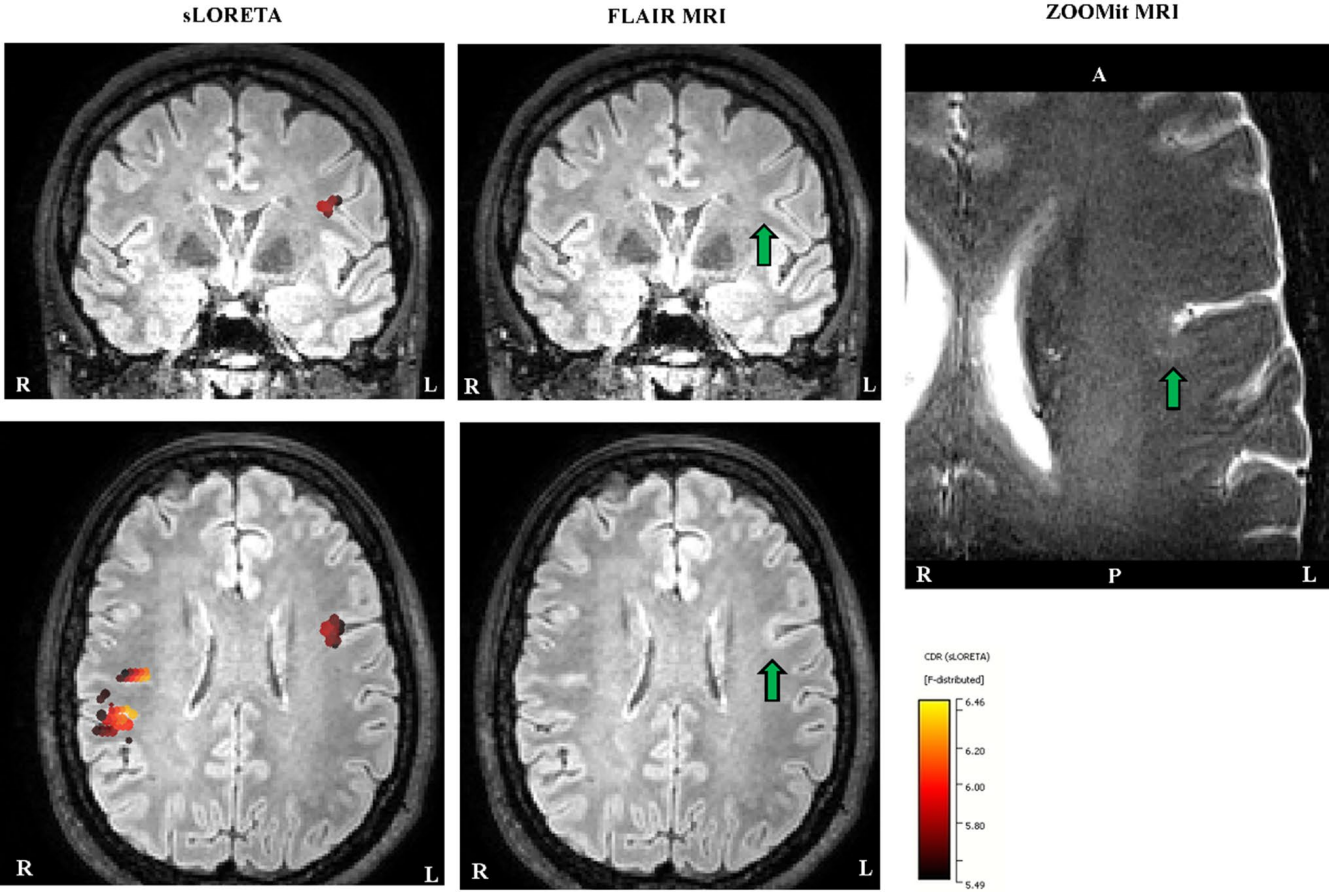
EMEG source localizations at −23 ms, slices selected according to the left hemispheric activity. In the left column, the sLORETA results registered to the FLAIR MRI are shown (only results obtained with a threshold of 85% for the maximum F-value are shown). In the middle column, the FLAIR image without localizations is shown, in order to enable the identification of the FCDs (pointed to by *green arrows*). The right column shows the ZOOMit MRI for the localization cluster (detected with sLORETA) at the left fronto-central region with the *green arrow* indicating the FCD. *White letters* on the MRIs show the directions (*L* left, *R* right, *A* anterior, *P* posterior). (Color figure online)

**Fig. 8:**
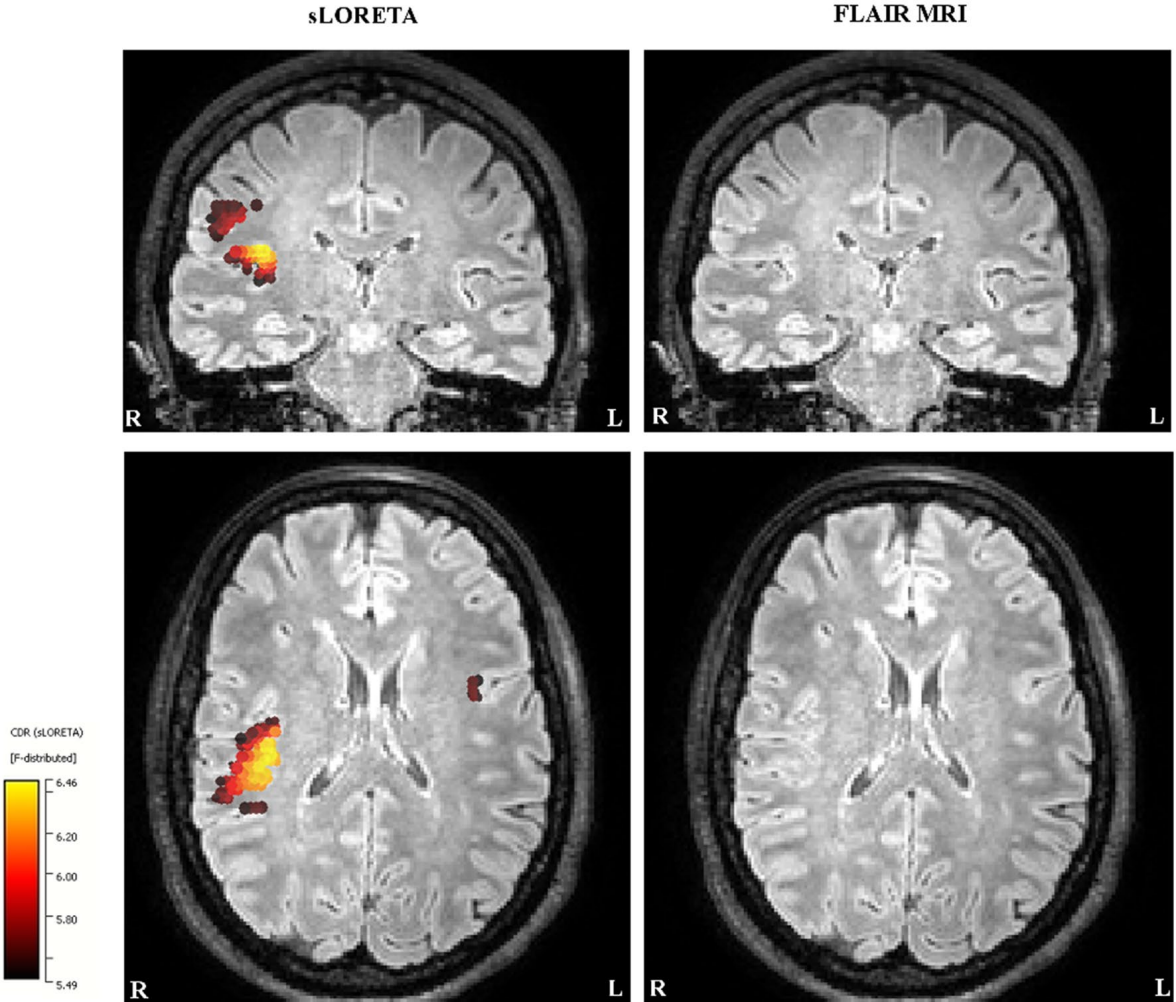
EMEG source localizations at −23 ms, slices selected according to the right hemispheric activity. The sLORETA results registered to the FLAIR MRI are shown in the left column (only results obtained with a threshold of 85% for the maximum F-value are shown). In the right column, the FLAIR image without the localizations is shown. The *white letters* on MRIs indicate the direction (*L* left, *R* right, *A* anterior, *P* posterior)

The left column in Fig. 6 shows the sLORETA reconstruction at −7 ms, projected onto the FLAIR MRI. The activity was localized in the right frontal region (note the radiological convention of L and R in Figs. 6, 7 and 8: patient’s left is viewer’s right). The location was concordant with an FCD that was detected on the FLAIR MRI prior to source analysis (pointed at by the green arrow in Fig. 6, middle column). The high resolution ZOOMit MRI of this ROI is shown at the right column. Note the clear improvements in detecting the boundary of the FCD with the ZOOMit sequence.

Usually, a clear FCD in FLAIR supported by interictal EEG source analysis showing the involvement of the area surrounding the FCD during IEDs would have been sufficient to decide on surgery. However, in this case the seizure semiology was not in concordance with the right frontal FCD and source reconstruction near the spike peak. The seizure semiology, involving tingling feeling at the right anterior torso, ascending feeling of nausea, then loss of consciousness and hypermotor movement of right arm and leg, was pointing to left frontocentral regions where MRI postprocessing had shown a potential second FCD.

### EMEG Source Analysis Prior to Spike Peak and ZOOMit MRI of a Second ROI

In order to check if the mismatch between the sLORETA results near the peak of the spike, MRI and seizure semiology was due to propagated activity, we have investigated time points prior to the spike peak. As mentioned before, we noticed another dipolar pattern at the time of the preceding MEG peak (−23 ms) in Figs. 4 and 5.

Performing source analysis at −23 ms highlighted two activity clusters: a focal one in the left frontocentral region (see left column in Fig. 7), and more dispersed activity in the right central region (see left column in Fig. 8). Being in agreement with the seizure semiology, the left frontocentral cluster was particularly intriguing. Visually reinvestigating, the FLAIR MRI at this ROI still remained negative (see arrows in Fig. 7, middle column). However, the ZOOMit MRI of that ROI (160 × 82 × 28 mm^3^) revealed a small FCD (in accordance with MRI morphometry) at the location pointed to by EMEG source analysis (see the arrow in Fig. 7, right column).

We did not find any abnormality near the location of the right central cluster in Fig. 8.

### DTI Tractography

**Fig. 9:**
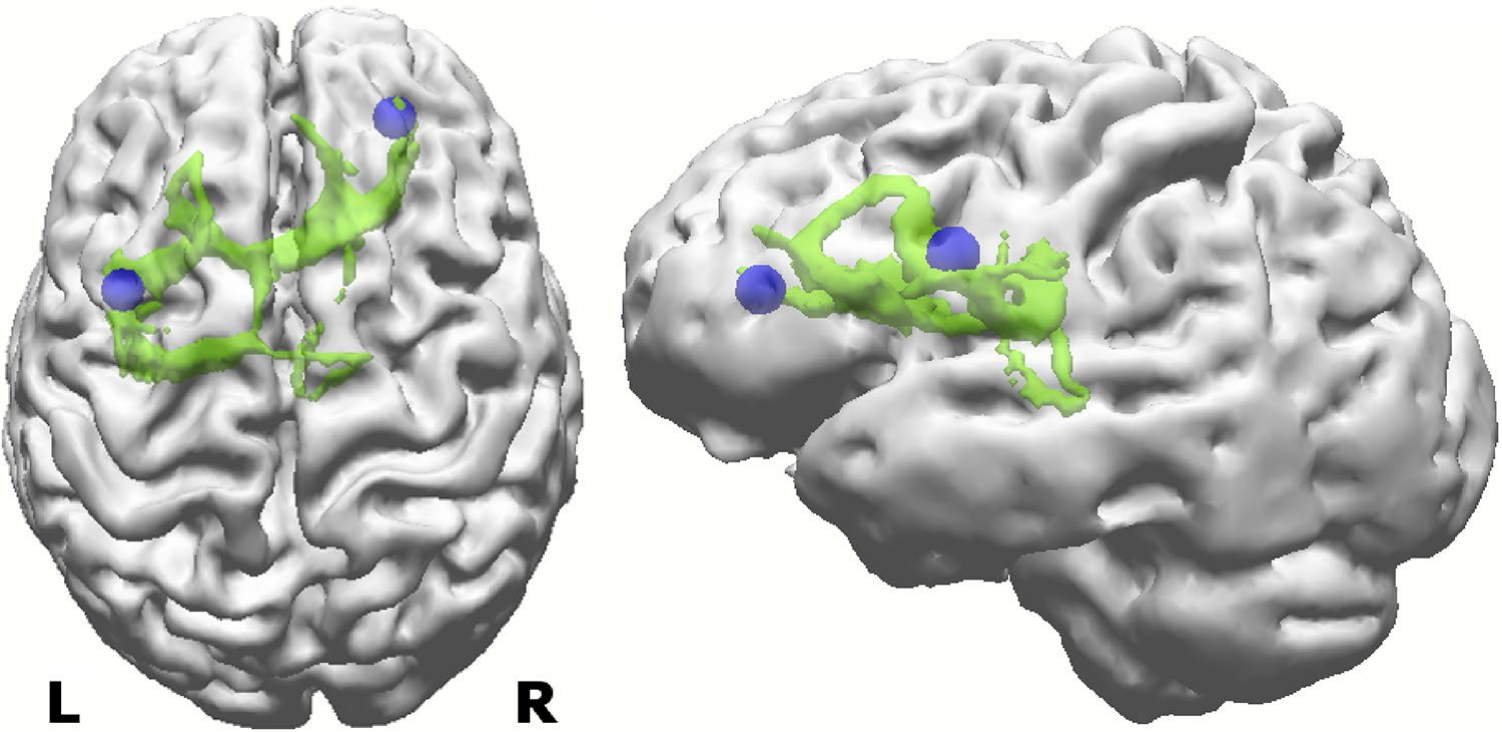
The results of DTI tractography. The *green paths* show the tracts that were found between the two FCDs indicated by *blue spheres*. (Color figure online)

Figure 9 illustrates the main tracts (green paths) that were found between the two FCDs (blue spheres) by DTI tractography and which might suggest a possible anatomical pathway between these two FCDs.

### Differences Between EEG, MEG, and EMEG Source Analysis

**Fig. 10:**
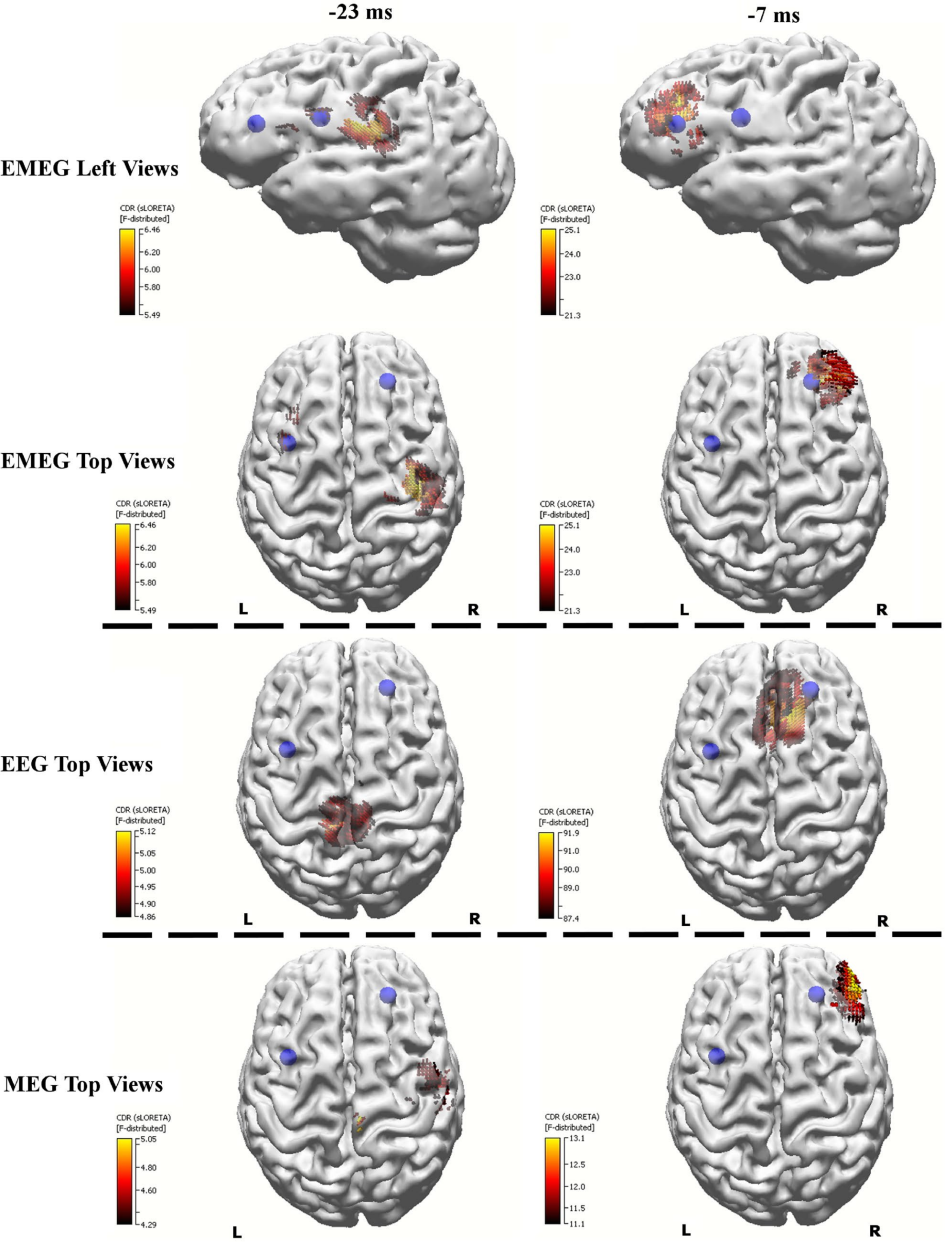
EMEG (*top two rows*), EEG only (*third row from the top*) and MEG only (*bottom row*) based source reconstructions at −23 ms (*left column*) and −7 ms (*right column*). A threshold of 85% of the maximum F-value was used for the results. The FCDs detected with MRI are indicated by the *blue spheres*. (Color figure online)

So far only the results of EMEG source analysis were presented. Figure 10 demonstrates the differences among EEG, MEG, and EMEG source analysis. The results at −23 ms (left column) and at −7 ms (right column) are shown superimposed to 3D volume rendering of the individual brain and the positions of the FCDs are indicated with blue spheres. For EMEG (top 2 rows) the results are very concordant with the positions of FCDs (with right frontal FCD at −7 ms and with left frontocentral FCD at −23 ms). EEG only (3rd row) and MEG only (bottom row) source analysis at −7 ms still localizes somewhat to the vicinity of the right frontal FCD, although with less agreement as compared to EMEG. Interestingly, the EEG result at −23 ms (3rd row, left) was totally misplaced pointing at a much more posterior and medial region. MEG results at −23 ms (bottom row, left) were concordant with EMEG in the right hemisphere but totally missed the activity near the left frontocentral FCD.

## Discussion

We studied a new multimodal approach in presurgical epilepsy diagnosis that benefits from (i) combined information from EEG and MEG, (ii) an individual high resolution finite element head model, (iii) individually calibrated skull conductivity, and (iv) recent advancements in morphometric MRI analysis and multi transmit and receive head coils. The first step was simultaneous EEG/MEG recordings followed by an MRI session acquiring T1w, T2w, DTI and FLAIR data with typical resolutions (1.875 mm edge length for DTI and 1.17 mm for the rest). This first study MRI session and the combined somatosensory evoked potential and field data were used to construct the calibrated finite element head model and thus solving the forward problem of EEG/MEG. Combined EEG/MEG (EMEG) source analysis was then performed using a distributed source approach to calculate the active areas in the brain, close to the peak of the averaged interictal epileptic discharges, as well as at earlier phases. Later, a second MRI session was performed, this time, using a new zooming technique (ZOOMit) to acquire high resolution images (0.5 mm voxel edge length) within two limited regions. These regions were selected based on EMEG source analysis near the peak (right frontal region) and at an earlier phase (left frontocentral region). The ZOOMit MRI revealed one relatively clear FCD at the right frontal region, and another subtle FCD at the left frontocentral region. Of interest, the left frontocentral FCD was not identifiable in 3D-FLAIR and only this one, and not the right frontal FCD, was concordant with seizure semiology. The second FCD was not detected in visual evaluation of any previous clinical MRIs at 3 T acquired and investigated in different centers; not even retrospectively. DTI tractography suggested a possible anatomical pathway supporting a fast propagation from the left frontocentral to the right frontal FCD. Further converging evidence for the hypothesis, although not very clear, was obtained from the morphometric MRI analysis (Huppertz et al. 2005) following an epilepsy specific protocol (Wellmer et al. 2013). The morphometric analysis hinted at a suspicious area, among others, close to left frontocentral FCD. Based on this converging evidence only the left frontocentral FCD had been treated using stereotactic radiofrequency thermocoagulation and the surgical outcome as well as the intracranial EEG supported our diagnosis (see Fig. 1 and the “Patient” section).

In addition to the cluster in the left hemisphere, due to quite clear further activity in MEG and EEG (see Mspikes topographies in Figs. 4, 5), we also found a cluster in the right hemisphere at −23 ms. This cluster was not investigated further because it was not in concordance with the seizure semiology. However, it is possible that the right central region was involved in interictal spikes as well but not in seizure generation. Although, irritative and seizure onset zones usually coincide it has been shown that this might not always be the case. For example, for some patients with bi-temporal spikes (with irritative zones in both right and left hemispheres) seizure freedom was achieved after performing operation in just one of the temporal lobes (Lüders et al. 2006). This also points to the importance of not using just one type of information but performing a multimodal strategy in presurgical epilepsy diagnosis.

As mentioned in the “Introduction” section there have been significant advances in the field of EEG/MEG source analysis in recent years, and multimodal combination of other noninvasive techniques, such as MRI, with source analysis could provide a promising way to increase the specificity. FCDs are intrinsically epileptogenic cortical malformations, resection of which leads to a high chance of seizure freedom (Sisodiya 2000). It has been reported that even in many MRI negative cases, post-operative histology could show an underlying FCD: (Lee et al. 2005) showed this number could be as high as 73%. In (McGonigal et al. 2007) histopathology showed FCD or hippocampal sclerosis for 12 out of 23 MRI negative patients, this number was 9 out of 29 in (Bien et al. 2009). Therefore, there is no doubt that many patients will benefit if the number of false negative MRIs could be reduced and the most obvious way to do that is going for higher spatial resolutions and better SNR, while keeping the examination time short enough to avoid patient movement. The main advantage of the here proposed ZOOMit technique was that it benefitted from localized excitation utilizing 2D selective RF pulses (Finsterbusch 2010) with parallel transmission (Blasche et al. 2012). As mentioned in the methods section localized excitation allows to ‘zoom’ a field of view, restricting excitation to a desired area even within brain tissue without aliasing artifacts that occur when the FOV is smaller than the imaged object. This avoids the need to increase the number of phase encoding steps and the penalty of an increased minimum measurement time. This localized excitation combined with fine tuned contrast parameters allowed us to obtain a combination of T2- and T1-weighting with increased lesion visibility and high resolution using a 3 T MRI.

FCDs can be difficult to identify with MRI and vice versa, ambiguous structural alterations may falsely be regarded as FCDs. Especially subtle findings may therefore not always imply significance for seizure generation. As we have shown regarding the right frontal FCD in this patient, the epileptic focus responsible for the seizures might even be far away from it. It is possible that the patient studied here has two potential epileptic foci and the right frontal one is pharmacosensitive; therefore, we did not record seizures from there. The study of (Brodbeck et al. 2010) showed the importance of source localization in MRI negative cases and (Zhang et al. 2014) presented an important review on the increasing value of multimodal imaging in epilepsy.

Although indirectly, in this study we have also demonstrated the importance of EMEG source analysis for the planning of intracranial electrode placement. In this study, if the intracranial electrodes had been placed near the right frontal FCD alone, the earlier epileptic activity arising from the left frontocentral FCD could not have been measured (because invasive electrodes are only sensitive to activity from close proximity). This would probably have led to the implantation of a second set of intracranial electrodes, based on seizure semiology, in order to detect the left frontocentral focus. This indicates the importance of tailoring the implantation of invasive electrodes by combining the information obtained from noninvasive EEG/MEG, MRI and the seizure semiology. In this direction (Knowlton et al. 2009) and (Agirre-Arrizubieta et al. 2014) have also shown the importance of MEG in the placement of intracranial EEG.

Another important point was that only combined EEG/MEG source analysis was able to localize the activity at the epileptic focus (activity near the left frontocentral FCD). Even though single modality EEG or MEG source analysis also detected activity very similar to the EMEG near the peak of the spike (localization of the right frontal FCD), their results were far away from the epileptic focus, which was only detectable near the spike onset. The topographies in Figs. 4 and 5 show hints of a left central dipolar pattern for both EEG and MEG. However, the SNR at this time instant was considerably too low and we think this is the reason for seeing the left frontocentral localization only in EMEG. This result is in line with previous studies showing the advantages of combined EEG/MEG in comparison to single modality source analysis because of the more stable source reconstructions and the superior spatial resolution (Cohen and Cuffin 1987; Fuchs et al. 1998; Baillet et al. 1999; Huang et al. 2007; Aydin et al. 2014, 2015; Chowdhury et al. 2015; Lucka 2015). In one of our previous studies we could show that the complementary information of EEG and MEG is especially important when the SNR of the data is low, such as at the spike onset (Aydin et al. 2015). In (Aydin et al. 2015) and (Chowdhury et al. 2015) it was also shown that the EMEG localizations were not simply the union of EEG and MEG results but a rather complicated interplay of both modalities compensating their relative shortcomings.

In this study, based on the results of our previous work (Aydin et al. 2015), we used an advanced realistic head model with seven different tissue compartments and white matter anisotropy modeled from DTI. However, we are aware that the generation and use of such a realistic head model might often not be feasible in clinical routine work and its clinical value will have to be evaluated in a larger series of patients. In such cases, following the findings of (Aydin et al. 2014; Vorwerk et al. 2014), we would suggest calibrating the skull conductivity and adding CSF and white/gray matter distinction which requires overall less effort, but still could considerably improve the results, and could especially enable combined EEG and MEG source analysis.

The main aim of this work was the presentation of a proof-of-principle, i.e., the methodology for a new multimodal presurgical epilepsy diagnosis approach and its feasibility and success in a case study with a multi-focal epilepsy patient that suffered from pharmaco-resistant focal onset epilepsy for 47 years of her life. As an outlook, the most important future goal will now be the reproduction of the presented results in a study with a larger group of epilepsy patients, a goal, which might not only be tackled by our working group, since methodology for combined EEG/MEG source analysis and MRI scanners using parallel transmit technology are now becoming more and more available.

## Conclusions

We presented a new methodology pipeline that combines the information from EMEG source analysis and recent advancements in MRI technology. The main novelty of this study is the use of a zoomed MRI technique (ZOOMit) to acquire high resolution (0.5 mm voxel edge length) data from an EEG/MEG prelocalized small region of interest, with good SNRs and reasonable acquisition times (13 min). To the best of the authors’ knowledge the proposed combined EMEG-zoomed MRI methodology is a new diagnosis strategy that has not been reported before. The methodology proved its value by detecting a small FCD, which otherwise was not detectable with standard MR sequences, within the epileptic focus. The main findings of this study were: (i) combined EMEG source analysis performs better than EEG or MEG alone, especially at lower SNRs, (ii) although EMEG source analysis at the peak of IEDs was localized well to an FCD this was only a propagation zone and not the real generator, (iii) only the converging evidence from combined EMEG source analysis at the spike onset, seizure semiology, and zoomed MRI was sufficient to identify the real generator.

## Footnotes

1 Seg3D: Volumetric Image Segmentation and Visualization. Scientific Computing and Imaging Institute (SCI), downloaded from: http://www.seg3d.org/.

2
http://www.rheinahrcampus.de/~medsim/vgrid/index.html.

3
http://www.mrt.uni-jena.de/simbio, the integration into Fieldtrip: http://fieldtrip.fcdonders.nl/development/simbio).

4
http://www.neuroscan.com/curry.cfm.
